# A novel ablation strategy of premature ventricular contractions originating from summit guided by CartoUNIVU module

**DOI:** 10.1002/clc.23390

**Published:** 2020-05-19

**Authors:** Xuexun Li, Jianping Li, Hongxia Chu, Xingpeng Liu

**Affiliations:** ^1^ Department of Cardiology Capital Medical University Beijing China; ^2^ Department of Cardiovascular Qingdao University Medical College Affiliated Yantai Yuhuangding Hospital Qingdao China; ^3^ Department of Cardiology Beijing Chaoyang Hospital, Capital Medical University Beijing China

**Keywords:** catheter ablation, left ventricular summit, premature ventricular contraction, ventricular arrhythmia

## Abstract

**Background:**

Premature ventricular contractions (PVCs) from left ventricular (LV) summit remain challenging for the risk of coronary artery injury. Computed tomographic or intracardiac echocardiography may be helpful, but both still have many limitations. CartoUNIVU module has rarely been used in PVC ablation.

**Methods:**

A total of 22 patients (14 men: mean age 56.4 ± 13.3 years) with an electrocardiogram indication of summit PVCs were included in the two centers study. A novel strategy ablation with the Image Integration Module CartoUNIVUTM module was performed for all the patients with PVCs originating from LV summit area, especially to prevent the coronary artery injury.

**Results:**

The procedure time was 78.6 ± 22.7 minutes, and the fluoroscopy time was 12.5 ± 3.1 minutes. The distance between the target and nearest coronary artery was 8.0 ± 3.1 mm. Three patients with the distance to the nearest coronary artery <5 mm. During a mean follow‐up of 11.0 ± 1.7 months, 21/22 (95.5%) patients were free from clinical PVC. No coronary artery injury was detected in the all the ablation procedures.

**Conclusion:**

The novel strategy ablation with the Image Integration Module CartoUNIVU module is safe and effective for PVCs originating from LV summit area, especially to prevent the coronary artery injury.

## 1 INTRODUCTION

Recently, a great progress has been made in electrophysiology and devices, which could reveal a detailed cardiac anatomy and improve outcomes of ablation of ventricular arrhythmia (VAs).^1^, ^2^, ^3^ The prevalence, electrocardiogram (ECG), and electrophysiological characteristics of idiopathic VAs have already been confirmed by many studies.^4^, ^5^, ^6^, ^7^, ^8^ However, owing to the complex anatomical structures like thick epicardial fat pads and proximity to the coronary arteries, the ablation of VAs originating from left ventricular (LV) summit remain challenging.^9^, ^10^, ^11^ The most serious complication involved with the ablation procedure is coronary artery injury. Integration of 3D electroanatomical mapping with computed tomographic (CT) or intracardiac echocardiography (ICE), CartoUNIVU module are increasingly used during ablation procedures and allow real‐time visualization of cardiac structures.^12^, ^13^, ^14^, ^15^

However, the CT and ICE have limitations to identify the detailed anatomical relations between the coronary artery and the target area of summit premature ventricular contraction (PVC). In our study, we would introduce a novel strategy with CartoUNIVU module to facilitate ablation PVC arising from LV summit and avoid coronary artery injury.

## 2 METHODS

Between January 2018 and March 2019, patients from Yantai Yuhuangding Hospital and Beijing Chaoyang Hospital with symptomatic PVCs indication for catheter ablation were enrolled in this retrospective study. The originates of the PVCs were identified in the summit area by the 12‐lead ECG and electroanatomical activation mapping. Antiarrhythmic medicine was discontinued for at least five half‐lives before the procedure. Transthoracic echocardiography was performed for all the patients prior to the procedure. The study protocol was approved by the ethic committee. All patients provided written informed consent.

### 2.1 CartoUNIVU module registration and coronary angiography

Patients were brought to the electrophysiology laboratory in the fasting, nonsedated state. Femoral vein and artery were both punctured for all the patients, and two 8‐F vascular sheaths were placed respectively. A decapolar catheter (Biosense‐Webster Inc., Irvine, California) with 2‐mm interelectrode distance was placed after subclavian vein puncture was performed. CartoUNIVU module is an advanced image integration module developed by Biosense Webster and could integrate the fluoroscopic images into 3D electroanatomical mapping systems (EAMS). This module is composed by software component connecting the CARTO system and X‐ray system, and by registration plate (RP). Before the registration process, a fluoroscopic image must be captured first with the fluoroscopy arm placed over the RP in the anterior‐posterior orientation. This image would be delivered to the CARTO system and the table is repositioned. Then, fluoroscopy arm projecting the heart could acquire standard views, and the registration process of CartoUNIVU module was performed. Coronary angiography from different angles like RAO30°, LAO30°, CAU20°, and RAO30°, LAO30°, and CRA20° were finished immediately after the registration process. Isoproterenol infusion would be used in the case of no spontaneous PVC.

### 2.2 Image integration module

After the registration process was performed, fluoroscopy images captured in different projections would be sent to the CARTO 3 system immediately. Then, all the fluoroscopy images could be integrated into the EAMS. The system could allow a 180° range divided into predefined areas of 5° for the right and left anterior oblique rotation. A 90° range divided into predefined regions of 5° could be allowed by the system for the caudal/cranial rotation. The EAMS would accurately display the intracardiac catheter position and the electroanatomical map could be created with the context of the captured images.

### 2.3 Mapping and ablation

An open irrigated 4 mm tip catheter (Smart‐Touch, Biosense‐Webster Inc.) was introduced to create the anatomical shells of great cardiac vein (GCV) (transvenous approach), aortic root, and LV (retrograde aortic approach) with the fluoroscopy image displayed on the map viewers.

During mapping in the coronary cusp region and LV, intravenous heparin was administered to achieve an activated clotting time ≥250 seconds. The target area where the PVC originates is determined after detailed activation mapping of all adjacent structures is finished. If the activation mapping is not satisfactory, a pace mapping would be applied.

Once the target area is determined, a distance between the target and the nearest coronary artery was measured on the merged image of coronary angiography and anatomical shells using the caliper of CARTO 3 system directly. If the distance is above 5 mm, the radiofrequency energy would be delivered. Maximum power delivery was 60 W and 35 W for the retrograde aortic and GCV approach, respectively. If the GCV approach cannot be performed, anatomical ablation from adjacent structures will be applied.

Successful catheter ablation was defined as the absence of spontaneous or inducible clinical PVC with isoproterenol infusion for 30 minutes after the ablation procedure. Coronary artery angiography was performed for all patients at the end of the procedure.

### 2.4 Follow‐up

Antiarrhythmic drugs were discontinued if the ablation procedure was successful. Postprocedure, all patients underwent a 24‐hour ECG monitoring. All the patients were followed in outpatient clinics. Symptoms and PVCs burden were assessed 6 or 12 months after discharge using 24‐hour Holter.

## 3 STATISTICAL ANALYSIS

Continuous variables were represented by mean ± SD, and categorical variables are expressed as numbers or percentages. An independent student *t* test was used for the continuous variables if necessary. Statistical analysis was performed with the SPSS 19.0 statistical package (SPSS Inc., Chicago, Illinois).

## 4 RESULTS

**FIGURE 1:**
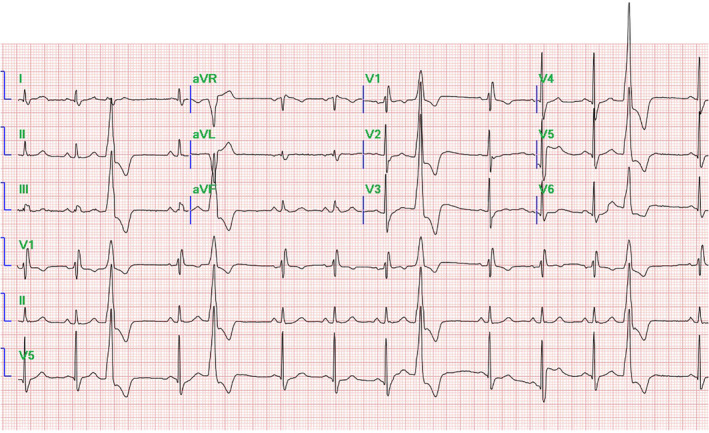
Electrocardiography of premature ventricular contraction (PVC) from left ventricular (LV) summit

**FIGURE 2:**
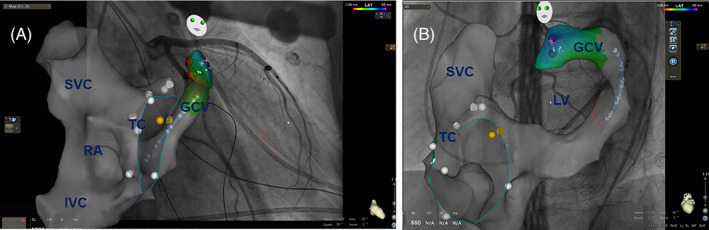
Integration of fixed fluoroscopy images into the 3D electroanatomical mapping system. A, RAO30° and, B, LAO45°. IVC, inferior vena cava; GCV, great cardiac vein; RA, right atrium; SVC, superior vena cava; TC, tricuspid valve

A total of 22 patients (14 men: mean age 56.4 ± 13.3 years) with an ECG indication of summit PVC underwent successful ablation (Figure 1). The LVEF of patients included in the study was 62.7 ± 4.4. Six patients had a failure ablation history. Baseline characteristics of the patients are represented in Table 1. The CartoUNIVU module was used in all cases to identify the distance between the target and nearest coronary artery (DTC) (Figure 2). The procedure time was 78.6 ± 22.7 minutes, and the fluoroscopy time was 12.5 ± 3.1 minutes. The distance between the target and nearest coronary artery was 8.0 ± 3.1 mm. The detailed successful ablation area is showed in Table 2. However, it should be noticed that three patients with the DTC < 5 mm (GCV 2, LCC 1). All the ablations were performed safely with a high power from the corresponding endocardium. Coronary artery angiography was performed for all patients at the end of the procedure, and no coronary artery injury was detected. The procedural data are represented in Table 2.

**TABLE 1:** Basic characteristics of the patients in the study

Sample size	22
Age, y	56.4 ± 13.3
Male, n (%)	14 (63.6%)
Hypertension, n (%)	10 (45.5%)
CAD, n (%)	7 (46.7%)
LVEF (%)	62.7 ± 4.4
Ablation failure history, n (%)	6 (27.3%)

Abbreviations: CAD, coronary artery disease; LVEF, left ventricular ejection fraction.

**TABLE 2:** Procedural characteristics

Sample size	22
Procedural time, min	78.6 ± 22.7
Fluoroscopy time, min	12.5 ± 3.1
Ablation time, min	7.5 ± 3.1
Distance between the target and coronary artery, mm	8.0 ± 3.1
Successful ablation area	
GCV, n (%)	3 (13.6)
LCC, n (%)	6 (27.3)
LV, n (%)	4 (18.2)
AMC, n (%)	7 (31.8)
LCC‐RCC commissure, n (%)	2 (9.1)
Ablation success, n (%)	22 (100)

Abbreviations: AMC, aortic mitral commissure; GCV, giant cardiac vein; LCC, left coronary cusp; LV, left ventricular.

### 4.1 Follow‐up

During a mean follow‐up of 11.0 ± 1.7 months, 21/22 (95.5%) patients were free from clinical PVC. The patient with a recurrent PVC was performed the ablation in the GCV in the prior procedure. After 3 months, he received another high power ablation from the corresponding endocardium side, and no PVC was detected after the second procedure.

## 5 ADVERSE EVENTS

All the patients were performed the echocardiography and ECG during the day after the procedure. No pericardial tamponade or effusion was found. No coronary artery injury was detected either.

## 6 DISCUSSION

This is the first study to perform the summit PVCs ablation with the Image Integration Module CartoUNIVU.

It proved that this novel approach is safe and effective and can avoid coronary artery injury caused by ablation. A high power ablation application can eliminate PVC originating from the epicardial summit area through which corresponding endocardium area.

LV summit is an area surrounded by the left anterior descending coronary artery, the first septal perforating branch, and the left circumflex coronary artery. VAs originating from the basal LV summit is still challenging because of thick epicardial fat pads and close proximity to coronary arteries.^1^, ^16^, ^17^ In some cases, it is unavailable even through transpericardial approach.^18^ In these cases, an anatomical ablation approach would be applied in our study, and this approach was very effective. For instance, if the earliest activation site was located in the anterior epicardial vein and ablation energy cannot be delivered, anatomical ablation was performed from adjacent structures like LCC or LV endocardium. Besides, maximum power delivery could be as high as 60 W in many cases. Shirai et al^19^ recently reported the efficacy of anatomical approach for outflow tract ventricular arrhythmias linked to the coronary venous system. This study specially analyzed the factors affecting the outcomes with this approach. It revealed that the main factor determining the successful ventricular arrhythmias elimination with anatomical approach was the anatomical distance between the coronary venous system site and the targeted adjacent site, and a cut‐off distance of >12.8 mm strongly predicted failure ablation.

The PVC arising from LV summit can easily be identified by the ECG characterized by an RBBB pattern, great R‐wave amplitude in the inferior leads, smaller aVL/aVR and III/II ratios, later precordia transition, shorter QRS duration, MDI ≥0.55.^16^

Owing to complex anatomical constraints, the approaches to introduce the catheter to this area varied, like by aortic retrograde, GCV, atrial septal puncture, pericardial puncture.^20^ However, no matter which approach was applied, the identical location and distance between the coronary artery and target area cannot be fully displayed. Due to the visual inspection is not accurate and poor, many ablation procedures failed or caused serious complications. Although it has been reported that ICE can clarify the anatomic relationship between the coronary artery and the target, its operation is complex, and the anatomic relationship between the target and the coronary artery cannot be fully revealed from multiple angles, which still has many limitations.^21^

The approach reported in our study can fuse the coronary angiography and 3D mapping shell in real time (Supplementary Material, Video S1). It not only can accurately clarify the adjacent relationship between the target and the coronary artery, but also use the caliper with Carto to accurately measure the distance between the target and the coronary artery. During the ablation, the distance between the catheter and the coronary artery can be observed in real time, which effectively ensures the safety of the operation and avoids the risk of coronary artery injury. Compared with traditional approach, this strategy could significantly improve ablation outcomes like procedure time and fluoroscopic time. In a recent study of successful ablation VAs linked to the coronary venous system with traditional approach,^19^ the procedure time and fluoroscopic time were 342 ± 98 minutes and 45.0 ± 21.5 minutes. In our study, this novel strategy consumed much fewer time, 78.6 ± 22.7 minutes and 12.5 ± 3.1 in procedure time and fluoroscopic time, respectively.

We believe this method could also be helpful in other kinds of VAs ablation to avoid coronary artery injury.

### 6.1 Limitations

This is a retrospective two‐center study and the sample size is small. A 24‐hour Holter monitoring was not performed on all patients. No controlled group was used in the study. In future, a larger sample size and multicenter, controlled trials are still necessary.

## 7 CONCLUSION

The novel strategy ablation of PVCs originating from LV summit area with CartoUNIVU™ module contributes to safeness and success of procedure by means of displaying anatomical structures and giving out relative information to assist operators in the process of mapping and ablation.

## CONFLICT OF INTEREST

The authors declare no potential conflict of interests.

## Supporting information


**Video S1** Xxxx
